# An Underwater Velocity-Independent DOA Estimation Based on Improved Toeplitz Matrix Reconstruction

**DOI:** 10.3390/s25071965

**Published:** 2025-03-21

**Authors:** Xuejin Zhao, Zihan Lei, Yu Wang, Gengxin Ning

**Affiliations:** 1 School of Economics, Guangdong University of Technology, Guangzhou 510006, China; zhaoxj@gdut.edu.cn; 2 School of Electronic and Information Engineering, South China University of Technology, Guangzhou 510641, China; 202120112318@mail.scut.edu.cn (Y.W.); ninggx@scut.edu.cn (G.N.)

**Keywords:** underwater DOA estimation, velocity independent, expanded coprime array, low-rank matrix reconstruction

## Abstract

Conventional acoustic velocity-independent direction of arrival (DOA) estimation models have limited measurement ranges and low degrees of freedom. This paper proposes an omnidirectional DOA estimation model based on improved Toeplitz matrix reconstruction to address these issues. The proposed method focuses on the Toeplitz matrix reconstruction method for sparse arrays to enhance the degree of freedom of the arrays. The method employs an expanding coprime array with a larger aperture, eliminating the acoustic velocity factor through geometric relationships and constructing a larger-size Toeplitz matrix. In addition, the concept of “low-rank matrix reconstruction” is introduced to fill the vacant terms in the Toeplitz matrix. Finally, the simulation experiments demonstrate the effectiveness of the proposed algorithm in improving the estimation accuracy.

## 1. Introduction

Underwater DOA estimation techniques have broad applications in marine detection, sonar localization, and underwater communication systems [1]. As acoustic waves propagate with lower attenuation than electromagnetic waves in underwater environments, acoustic signals are primarily used in underwater DOA estimation. The speed of sound in the ocean is typically between 1450 m/s and 1550 m/s [2]. Traditional DOA estimation methods assume a fixed speed of sound, which introduces inevitable system bias. Therefore, eliminating the impact of acoustic velocity errors is crucial.

Among the widely used DOA estimation techniques, the MUSIC algorithm [3] is known for its high-resolution performance, but suffers from a high computational complexity. To address this, numerous algorithms based on the ESPRIT algorithm [4] have been developed, including TLS-ESPRIT [5], Unitary ESPRIT [6], and MI-ESPRIT [7]. However, these algorithms treat the acoustic velocity as a constant, considering that the velocity is constantly changing in the underwater environment, using a fixed value instead of the real-time sound speed will lead to a large error. To address this issue, some velocity-independent algorithms have been proposed. The VI-MUSIC algorithm [8] uses the MUSIC spectrum from two uniform linear arrays (ULAs) to complete the estimation. The algorithm searches for acoustic velocity values within the 1450–1550 m/s range to minimize the difference between the two sets of estimates, thereby improving the DOA estimation accuracy. The VI-LC-ESPRIT algorithm [9] is based on the cross-linear array and utilizes the geometric properties of the rotation factors of the two line arrays to eliminate the acoustic velocity factor and estimate the DOA. Nishimura et al. [10] employed a non-uniform sensor array to simultaneously estimate the DOA and acoustic velocity through constrained nonlinear optimization. These algorithms are effective when the acoustic velocity is unknown, but they tend to suffer from larger estimation errors and narrow detection angles. The TVI-ESPRIT algorithm [11], which replaces the uniform array with a nested array and the covariance matrix with a large Toeplitz matrix, improves the estimation accuracy while maintaining the advantages of acoustic velocity independence and omnidirectional estimation.

Recently, sparse arrays have gained widespread attention. Sparse arrays offer notable advantages over ULA in terms of degrees of freedom, mutual coupling between elements, and redundancy. With the same number of physical array elements, sparse arrays achieve larger apertures and higher degrees of freedom, enabling the estimation of more signal sources, improving accuracy, and enhancing angular resolution. Typical sparse arrays include Minimum Redundancy Array (MRA) [12], Minimum Hole Array (MHA) [13], Nested Array (NA) [14], and Coprime Array (CA) [15]. These typical sparse arrays have inspired numerous new array design ideas [16,17,18], focusing on higher degrees of freedom and lower mutual coupling to improve the DOA estimation performance.

To address the issues faced by current underwater acoustic velocity-independent algorithms, such as estimation error and limitations in the detection angle, this paper proposes an underwater acoustic velocity-independent DOA estimation model based on an improved Toeplitz matrix reconstruction, referred to as the ECC-TVI-ESPRIT method. The proposed model builds upon the omnidirectional DOA estimation framework of TVI-ESPRIT, utilizing an expanded coprime array (ECA) with a larger array aperture and constructing a larger Toeplitz matrix. The concept of “low-rank matrix reconstruction” is introduced to fill in the vacant terms of the Toeplitz matrix. Finally, the ESPRIT algorithm is used to carry out the DOA estimation.

## 2. Models and Methods

### 2.1. Signal Model

**Figure 1:**
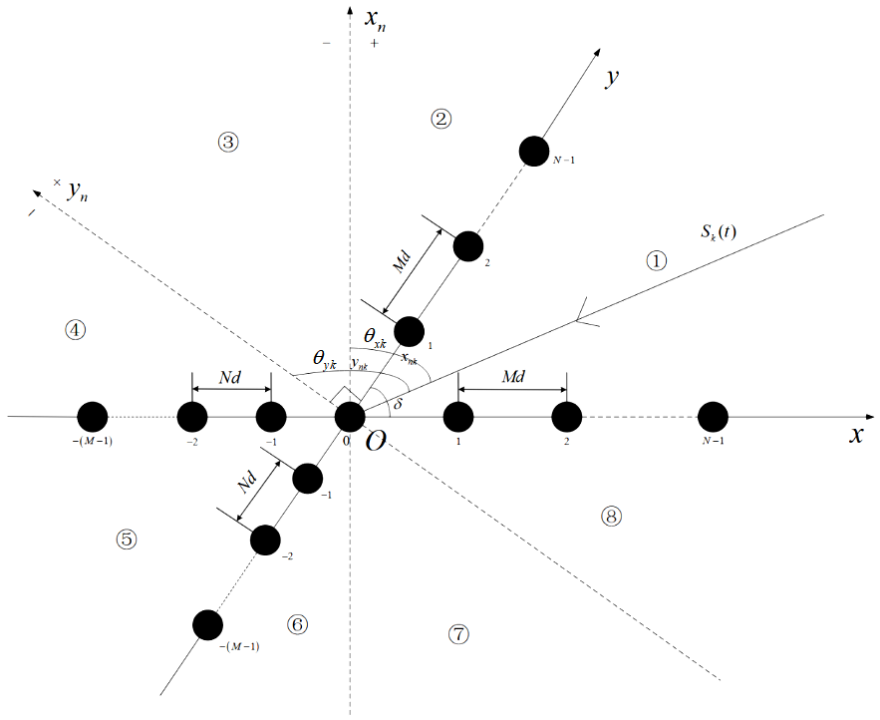
Arbitrary cross-expanded coprime array structure.

As shown in Figure 1, assume that the signal receiving array consists of two identical expanded coprime arrays, with a crossing angle denoted as δ. Each coprime array is composed of two subarrays, with *M* and *N* elements in each subarray, respectively.

Assume that there are *K* mutually uncorrelated far-field narrowband signals arriving at the cross- array. The arrival angle of the *k*th signal with respect to the *x*-axis array is denoted as $\theta_{xk}\left(k=1,2,\ldots ,K\right)$, and with respect to the *y*-axis array as $\theta_{yk}\left(k=1,2,\ldots ,K\right)$. Based on the omnidirectional signal estimation model proposed inthe TVI-ESPRIT algorithm, their relationship is expressed as follows:

(1) $$sin\theta_{yk}=sin(\theta_{xk}+\delta )\;k=1,2,\ldots ,K$$

The signal matrix in the spatial domain is denoted as S, where *L* represents the number of snapshots. The received signal matrices for the two arrays, $X,Y\in C^{(M+N)\times L}$ are given by:

(2a) $$\begin{array}{c} X=A_{x}S+N_{x} \end{array}$$

(2b) $$\begin{array}{c} Y=A_{y}S+N_{y} \end{array}$$

where $A_{x},A_{y}\in C^{(M+N)\times K}$ are the manifold matrices of the two line arrays and $N_{x},N_{y}\in C^{(M+N)\times L}$ are the Gaussian Noise matrices with a mean of 0 and variance $\sigma_{n}^{2}$.

The covariance matrices of the arrays are then computed as follows:

(3a) $$\begin{array}{c} R_{x}=E\left\{{\mathrm{XX}}^{H}\right\}=A_{x}R_{s}A_{x}^{H}+\sigma_{n}^{2}I_{n} \end{array}$$

(3b) $$\begin{array}{c} R_{y}=E\left\{{\mathrm{YY}}^{H}\right\}=A_{y}R_{s}A_{y}^{H}+\sigma_{n}^{2}I_{n} \end{array}$$

By vectorizing the matrices, we obtain the following:

(4a) $$\begin{array}{c} r_{x}=\mathrm{vec}\left\{R_{x}\right\}=(A_{x}^{*}⊙A_{x})p+\sigma_{n}^{2}1_{n} \end{array}$$

(4b) $$\begin{array}{c} r_{y}=\mathrm{vec}\left\{R_{y}\right\}=(A_{y}^{*}⊙A_{y})p+\sigma_{n}^{2}1_{n} \end{array}$$

where ⊙ represents the Khatri–Rao product, $p={\left[\sigma_{1}^{2},\sigma_{2}^{2},\ldots ,\sigma_{K}^{2}\right]}^{T}\in C^{K\times 1}$ is the power vector of the signal, and $1_{n}$ is the vectorized identity matrix.

For convenience, *M* is used to represent the number of elements in one array, instead of (M+N) above. At this point, the vectors $r_{x},r_{y}\in C^{M^{2}\times 1}$ can be regarded as the array signal of a single snapshot, and the corresponding virtual array element positions are the difference of the actual array element positions, as follows:

(5) $$S=\{l_{i}-l_{j}:l_{i},l_{j}\in L\}$$

where L is the actual element position distribution of the array.

Obviously, the arrangement of S has two main issues: the distribution of array elements is disordered and not in ascending order, and redundant signals exist across multiple elements. Therefore, a linear transformation matrix is required to perform sorting and eliminate redundancy, denoted as $F_{r}\in C^{\mathrm{dof}\times M^{2}}$, with dof representing the degrees of freedom of the differential array after processing. The mapping matrix $F_{r}$ is in one-to-one correspondence with the sparse array arrangement. The signal values at some positions are missing and need to be interpolated and completed using techniques such as “low-rank matrix reconstruction”.

### 2.2. Low-Rank Matrix Reconstruction

To map the signals of a single snapshot ${\bar{z}}_{x},{\bar{z}}_{y}\in C^{\mathrm{dof}\times 1}$ to a ULA, the first step is to insert zeros at the positions of the “signal holes”. This requires constructing a new 0–1 transformation matrix $H\in C^{(2M_{tp}-1)\times \mathrm{dof}}$, as follows:

(6a) $$\begin{array}{c} z_{lx}=H{\bar{z}}_{x} \end{array}$$

(6b) $$\begin{array}{c} z_{ly}=H{\bar{z}}_{y} \end{array}$$

where $z_{lx},z_{ly}\in C^{(2M_{tp}-1)\times 1}$ are the single snapshot signals after zero padding.

Then, using the single snapshot vectors $z_{lx},z_{ly}$, the $M_{tp}\times M_{tp}$ Toeplitz matrix $R_{xt},R_{yt}\in C^{M_{tp}\times M_{tp}}$ is constructed:

(7a) $$\begin{array}{c} R_{xt}=\left(\begin{array}{cccc} z_{lx}(M_{tp}) & z_{lx}(M_{tp}-1) & \cdots & z_{lx}(1)\\ z_{lx}(M_{tp}+1) & z_{lx}(M_{tp}) & \cdots & z_{lx}(2)\\ \vdots & \vdots & \ddots & \vdots \\ z_{lx}(2M_{tp}-1) & z_{lx}(2M_{tp}-2) & \cdots & z_{lx}(M_{tp}) \end{array}\right) \end{array}$$

(7b) $$\begin{array}{c} R_{yt}=\left(\begin{array}{cccc} z_{ly}(M_{tp}) & z_{ly}(M_{tp}-1) & \cdots & z_{ly}(1)\\ z_{ly}(M_{tp}+1) & z_{ly}(M_{tp}) & \cdots & z_{ly}(2)\\ \vdots & \vdots & \ddots & \vdots \\ z_{ly}(2M_{tp}-1) & z_{ly}(2M_{tp}-2) & \cdots & z_{ly}(M_{tp}) \end{array}\right) \end{array}$$

The corresponding holes in the Toeplitz matrix are filled with zeros, which requires performing a “matrix completion” operation.

The aperture of the uniform array mapped by this Toeplitz matrix is $M_{tp}$. With *K* incoherent signals in the spatial domain, the rank of the matrices $R_{xt},R_{yt}$ should be *K*, with dimensions of $M_{tp}$. Based on the basic assumptions of the DOA estimation model, the following relationship holds:

(8) $$M_{tp}-1\geq K$$

Therefore, $R_{xt},R_{yt}$ satisfy the definition of the low-rank matrix.

Let the completed Toeplitz matrices be denoted as $T_{x},T_{y}\in C^{M_{tp}\times M_{tp}}$. As the power $\sigma_{s1}^{2},\sigma_{s2}^{2},\ldots ,\sigma_{sK}^{2}$ of the signal in the spatial domain is always greater than or equal to 0, $T_{x},T_{y}$ are positive semi-definite Hermitian Toeplitz matrices.

(9a) $$\begin{array}{c} T_{x}⪰0 \end{array}$$

(9b) $$\begin{array}{c} T_{y}⪰0 \end{array}$$

Under this condition, it is necessary to find a feasible completion strategy that minimizes the rank of the Toeplitz matrix.

(10a) $$\begin{array}{c} \min_{T_{x}}\mathrm{rank}\{T_{x}\} \end{array}$$

(10b) $$\begin{array}{c} \min_{T_{y}}\mathrm{rank}\{T_{y}\} \end{array}$$

Minimizing the rank of a matrix is an NP-hard and non-convex problem, which is difficult to solve. Therefore, it is often solved by convex relaxation to the trace of the matrix. For $T_{x}\in C^{M_{tp}\times M_{tp}}$, as it is a Hermitian Toeplitz matrix with rank $r=\mathrm{rank}\left\{T_{x}\right\}<M_{tp}$, it has a unique Vandermonde decomposition:

(11) $$T_{x}=\sum_{k=1}^{r}p_{k}a(\theta_{k})a^{H}(\theta_{k})=A(\theta )PA^{H}(\theta )$$

where A is the Vandermonde matrix and P is the diagonal matrix. Taking the trace of this matrix yields the following:

(12) $$\mathrm{Tr}\{T_{x}\}=M_{tp}\sum_{k=1}^{r}p_{k}$$

This is similar to the $\ell_{1}$ atomic norm:

(13) $$\left|\right|z_{lx}{\left|\right|}_{A,1}=\sum_{k=1}^{r}p_{k}=\mathrm{Tr}\{T_{x}\}/M_{tp}$$

where the vector $z_{lx}\in C^{M_{tp}}$ is spanned by the space $\left\{a(\theta_{k})\right\}$ and the corresponding set of coefficients $\{p_{k}\},k=1,2,\ldots ,r$ is unique. Thus, the inf{·} operation can be ignored and the $\ell_{1}$ atomic norm is the sum of this set of coefficients.

Thus, the convex optimization problem becomes:

(14a) $$\begin{array}{c} \min_{T_{x}}\mathrm{Tr}\{T_{x}\} \end{array}$$

(14b) $$\begin{array}{c} s.t.\;T_{x}⪰0 \end{array}$$

Additionally, it is necessary to define binary matrices $G_{x},G_{y}\in C^{M_{tp}\times M_{tp}}$ to indicate the positions of the observations. The positions where $T_{x}$ is not a value of 0 are set to 1 and the remaining positions are set to 0:

(15) $$G_{x}(i,j)=\left\{\begin{array}{cc} 1, & R_{xt}(i,j)\neq 0\\ 0, & R_{xt}(i,j)=0 \end{array}\right.$$

Furthermore, the magnitude of the change in the original observation during the matrix interpolation is measured by the Frobenius norm, denoted as the F-norm, which is a commonly used convex function. It is often necessary to limit the fitting error:

(16) $$\left|\right|T_{x}\circ G-R_{xt}{\left|\right|}_{F}^{2}\leq \eta$$

where ∘ denotes the Hadamard product. It can also be incorporated as part of the objective function, using a regularization parameter μ to balance the atomic norm and the fitting error. Modify the optimization problem Equation (14a,b) as:

(17a) $$\begin{array}{cc} & \min_{T_{x}}\left|\right|T_{x}\circ G-R_{xt}{\left|\right|}_{F}^{2}+\mu \mathrm{Tr}\{T_{x}\} \end{array}$$

(17b) $$\begin{array}{cc} & s.t.\;T_{x}⪰0 \end{array}$$

By solving the above convex optimization problems, two completed Toeplitz matrices $T_{x}^{*},T_{y}^{*}\in C^{M_{tp}\times M_{tp}}$ with larger degrees of freedom are obtained to replace the original covariance matrices.

### 2.3. Proposed Method

After the reconstruction of the Toeplitz matrix, the ESPRIT algorithm is separately applied to the two arrays. The process is summarized as follows. Eigenvalue decomposition is performed on $T_{x}^{*},T_{y}^{*}$ to obtain the eigenvectors corresponding to the *K* largest eigenvalues. The signal subspaces $U_{sx},U_{sy}\in C^{M_{tp}\times K}$ are then constructed and divided into two submatrices:

(18a) $$\begin{array}{c} \left\{\begin{array}{c} U_{sx1}=U_{sx}(1:M_{tp}-1,:)\\ U_{sx2}=U_{sx}(2:M_{tp},:) \end{array}\right. \end{array}$$

(18b) $$\begin{array}{c} \left\{\begin{array}{c} U_{sy1}=U_{sy}(1:M_{tp}-1,:)\\ U_{sy2}=U_{sy}(2:M_{tp},:) \end{array}\right. \end{array}$$

Then, the phase rotation matrices $\Psi_{x},\Psi_{y}\in C^{K\times K}$ for the two linear arrays are calculated:

(19a) $$\begin{array}{c} \Psi_{x}=U_{sx1}^{†}U_{sx2}=Y_{x}^{-1}\Phi_{x}Y_{x} \end{array}$$

(19b) $$\begin{array}{c} \Psi_{y}=U_{sy1}^{†}U_{sy2}=Y_{y}^{-1}\Phi_{y}Y_{y} \end{array}$$

where $Y_{x},Y_{y}\in C^{K\times K}$ are unitary matrices, and $\Phi_{x},\Phi_{y}\in C^{K\times K}$ are diagonal matrices with diagonal elements representing the rotation factors of different signals between the array elements.

The eigenvalue decomposition of Equation (19a,b) yields the rotation factors $\left\{{\hat{\Phi }}_{xk}\right\}$ and $\{{\hat{\Phi }}_{yk}\},k=1,2,\cdots ,K$ of the two arrays in turn. According to the expression of the rotation factors, we have:

(20) $$\left\{\begin{array}{c} \arg({\hat{\Phi }}_{xk})=\frac{2\pi f_{0}d\sin{\hat{\theta }}_{xk}}{c_{k}}\\ \arg({\hat{\Phi }}_{yk})=\frac{2\pi f_{0}d\sin{\hat{\theta }}_{yk}}{c_{k}} \end{array}\right.$$

From this, we can obtain:

(21) $$\frac{\arg({\hat{\Phi }}_{yk})}{\arg({\hat{\Phi }}_{xk})}=\frac{\sin{\hat{\theta }}_{yk}}{\sin{\hat{\theta }}_{xk}}$$

It is evident from the above steps that the acoustic velocity factor is eliminated, thus removing the estimation errors caused by acoustic velocity. Finally, the DOA estimation of the *k*th signal is obtained by substituting Equation (1) into Equation (21):

(22) $${\hat{\theta }}_{k}=\left\{\begin{array}{cc} \mathrm{arccot}\left(\frac{\arg({\hat{\Phi }}_{yk})}{\arg({\hat{\Phi }}_{xk})\sin\delta }-\cot\delta \right), & \arg({\hat{\Phi }}_{xk})>0\\ \mathrm{arccot}\left(\frac{\arg({\hat{\Phi }}_{yk})}{\arg({\hat{\Phi }}_{xk})\sin\delta }-\cot\delta \right)-\pi , & \arg({\hat{\Phi }}_{xk})<0\\ 0, & \arg({\hat{\Phi }}_{xk})=0 \end{array}\right.$$

where the value domain of the arccot(·) operation is [0,π], which corresponds to the incident region {➀,➁,➆,➇} in Figure 1.

For the multi-source scenario, both sets of rotation factors $\left\{{\hat{\Phi }}_{xk}\right\}$ and $\left\{{\hat{\Phi }}_{yk}\right\}$ each contain *K* values, and a matching operation must be performed between them. The principle of minimizing the variance of the acoustic velocity estimation, i.e., assuming that the acoustic velocity difference between different array elements for a single signal is small. According to Equation (20), the estimate of the *k*th acoustic velocity can be obtained as follows:

(23) $${\hat{c}}_{k}=\frac{2\pi f_{0}d\sin\delta }{\sqrt{\arg^{2}\left({\hat{\Phi }}_{xk}\right)+\arg^{2}\left({\hat{\Phi }}_{yk}\right)-2\arg\left({\hat{\Phi }}_{xk}\right)\arg\left({\hat{\Phi }}_{yk}\right)\cos\delta }}$$

Finding the set of matches that minimize the variance:

(24) $$\min_{i}\mathrm{var}({\left\{{\hat{c}}_{k}\right\}}_{i}),\;i=1,2,\cdots ,K!$$

As the proposed velocity-independent algorithm is based on the Expanded Coprime Cross (ECC) array and uses Semidefinite Program (SDP) optimization to modify the Toeplitz matrix reconstruction, it is named ECC-TVI-ESPRIT method. The procedure is summarized in Algorithm 1.
**Algorithm 1** Framework of ECC-TVI-ESPRIT Algorithm**Input:**A cross-expanded coprime array with a crossing angle of δ, where each array consists of *M* elements.The received signal matrices $X,Y\in C^{M\times L}$.**Output:**1: Calculate the covariance matrices $R_{x}$ and $R_{y}$ of the two coprime arrays by Equation (3a,b), and vectorize them according to Equation (4a,b) to obtain $r_{x}$ and $r_{y}$.2: Sort and eliminate redundancy in the vectorized signals to obtain ${\bar{z}}_{x}$ and ${\bar{z}}_{y}$.3: Fill the signal holes with zeros using Equation (6a,b), and construct the Toeplitz matrix according to Equation (7a,b).4: Construct the binary matrices $G_{x},G_{y}$ using Equation (15), and solve the SDP optimization problem as Equation (17a,b) for two axes to obtain the solutions $T_{x}^{*},T_{y}^{*}$.5: Perform eigenvalue decomposition on $T_{x}^{*},T_{y}^{*}$ to obtain the signal subspace, then calculate the similar matrices $\Psi_{x}$ and $\Psi_{y}$ for the rotation factors by Equations (18a,b) and (19a,b).6: Estimate the acoustic velocity for each pairing by Equation (23) and identify the match set with the smallest variance.7: Compute the DOA estimation using Equations (22) and (24).8. **return** DOAs: ${\hat{\theta }}_{1},{\hat{\theta }}_{2},\ldots ,{\hat{\theta }}_{K}$.

In addition, taking the cross-array illustrated in Figure 1 as an example, assume that the number of elements along the *x*-axis is $M_{x}$, and the number of elements along the *y*-axis is $M_{y}$. The coordinates of the elements on the *x*-axis are denoted as $x_{m}\left(m=1,2,\ldots ,M_{x}\right)$, while those on the *y*-axis are $y_{m}\left(m=1,2,\ldots ,M_{y}\right)$. The Cramér–Rao Bound (CRB) of DOA estimation using the cross-array can be derived as:

(25) $$\mathrm{CRB}\left(\theta_{k}\right)=\frac{180^{\circ }}{\pi }\sqrt{\frac{c^{2}}{\omega^{2}L\gamma }{\left[cos^{2}\theta_{k}\sum_{m=2}^{M_{x}}x_{m}^{2}+cos^{2}\left(\theta_{k}+\delta \right)\sum_{m=2}^{M_{y}}y_{m}^{2}\right]}^{-1}}$$

where $\omega =2\pi f_{0}$ and γ represents the signal-to-noise ratio (SNR). The overall CRB for one-dimensional spatial signal estimation is given by:

(26) $$\mathrm{CRB}=\sqrt{\frac{1}{K}\sum_{k=1}^{K}{\mathrm{CRB}}^{2}\left(\theta_{k}\right)}$$

Based on Equations (25) and (26), it can be concluded that the CRB of 1D DOA estimation decreases as the SNR or the number of snapshots increases. Moreover, the crossing angle δ, the number of elements and their spatial configuration significantly impact the CRB. The optimal array configuration occurs when $\delta =90^{\circ }$ and $M_{x}=M_{y}$, where $M_{x}$ is the total number of elements in an array composed of two coprime subarrays. If the two subarrays contain *M* and *N* elements, respectively, the CRB reaches its minimum value when they satisfy the conditions for forming a coprime array.

### 2.4. Computational Complexity Analysis

Assume that the number of array elements is *M*, the number of snapshots is *L*, the number of signal sources is *K*, the size of the reconstructed Toeplitz matrix is $M_{tp}$, and the number of iterations for solving the SDP optimization problem is *W*.

For the TVI-ESPRIT algorithm, the complexity analysis is as follows: the complexity of obtaining the covariance matrix of the received signal is $O(2M^{2}L)$, vectorizing the matrix is $O(2M^{2})$, sorting and eliminating redundancy is $O(2\left(2M_{tp}-1\right)M^{2})$, constructing the Toeplitz matrix is $O(2M_{tp}^{2})$, the eigenvalue decomposition of the Toeplitz matrix is $O(2M_{tp}^{3})$, constructing the signal subspace and matrix division is $O(8\left(M_{tp}-1\right)K+2M_{tp}K)$, computing the rotation matrix is $O(4M_{tp}K^{2})$, the eigenvalue decomposition of the rotation matrix is $O(2K^{3})$, and matching the rotation factors is O(K·K!). Therefore, the overall complexity of the TVI-ESPRIT algorithm is $O(2M^{2}L+4M_{tp}M^{2}+2M_{tp}^{2}+2M_{tp}^{3}+10M_{tp}K+4M_{tp}K^{2}+2K^{3}+K\cdot K!)$.

For the ECC-TVI-ESPRIT algorithm, two additional complexity components are introduced: constructing the binary matrix G with a complexity of $O(2M_{tp}^{2})$ and solving the optimization problem (17) with a complexity of $O(6WM_{tp}^{2})$. Thus, the complexity of the ECC-TVI-ESPRIT algorithm is $O(2M^{2}L+4M_{tp}M^{2}+4M_{tp}^{2}+6WM_{tp}^{2}+2M_{tp}^{3}+10M_{tp}K+4M_{tp}K^{2}+2K^{3}+K\cdot K!)$.

## 3. Results and Discussion

This section evaluates the performance of the proposed ECC-TVI-ESPRIT algorithm by comparing it with TVI-ESPRIT, VI-ESPRIT, and Toeplitz-ESPRIT algorithms [14]. Specifically, the TVI-ESPRIT algorithm uses cross-nested arrays, the ECC-TVI-ESPRIT algorithm uses cross-expanded coprime array, the Toeplitz-ESPRIT algorithm uses nested linear arrays, and the VI-ESPRIT algorithm uses uniform cross-arrays.

To ensure the fairness of the experiments, the number of array elements is M=19 for all arrays. In addition, the crossing angle of the crossed arrays is set to $\delta =90^{\circ }$. The carrier frequency of all sources is $f_{0}=10$ kHz, the sampling frequency is $f_{s}=25$ kHz, and the number of snapshots is L=200. Each experiment is repeated with W=1000 Monte Carlo trials. Unless otherwise specified, the environment has an SNR of 0 dB with Gaussian white noise, and the incident angle are $\theta =30^{\circ }$ and $60^{\circ }$. The expected acoustic velocity is $c_{0}=1500$ m/s, and the unit spacing between the array elements is set to half of the expected signal wavelength. Let $\Delta c=c_{r}-c_{0}$, where $c_{r}$ is the true sound speed. The theoretical expression of the systematic error caused by inaccurate acoustic velocity is given by:

(27) $${\mathrm{Error}}_{c}=\left|\theta -\arcsin\left(\frac{c_{0}\sin\theta }{c_{r}}\right)\right|$$

As indicated by the above equation, when considering the acoustic velocity factor, the systematic error in DOA estimation increases as the acoustic velocity error grows.

### 3.1. Algorithm Validity Test

This set of experiments is designed to validate the DOA estimation performance of the ECC-TVI-ESPRIT algorithm. The ECC-TVI-ESPRIT and TVI-ESPRIT algorithms use the omnidirectional DOA estimation model, while the VI-ESPRIT and Toeplitz-ESPRIT algorithms use the traditional unidirectional DOA estimation model.

Three incoherent signals with a center frequency of 10 kHz are set, and their DOAs are $\theta =\{-75^{\circ },60^{\circ },165^{\circ }\}$. The actual underwater acoustic velocity is set to 1480 m/s, while the expected acoustic velocity is 1500 m/s, the SNR is 10 dB, and the number of snapshots is 200.

**Figure 2:**
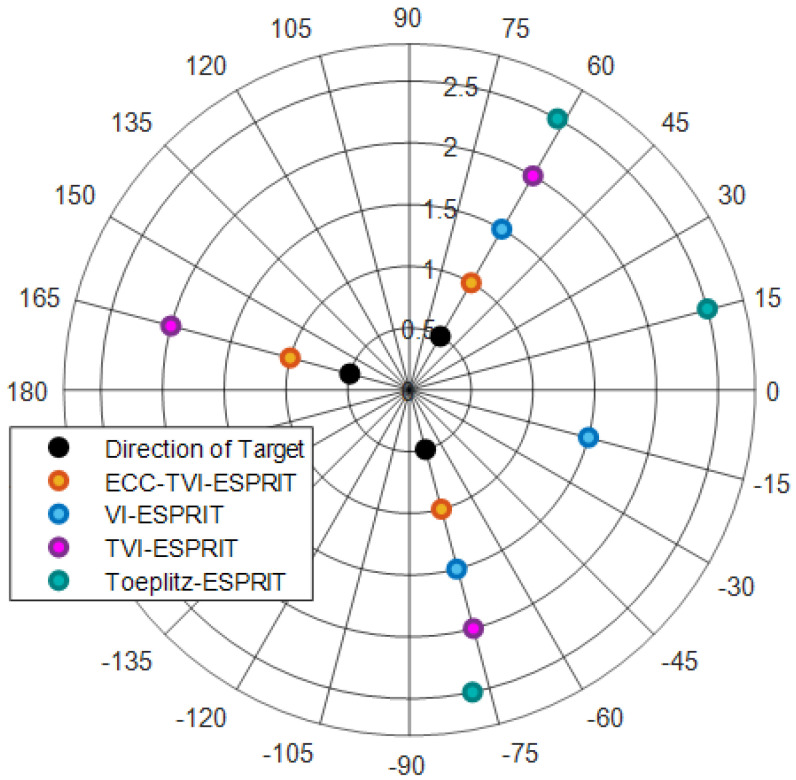
Omnidirectional DOA estimation.

Figure 2 shows that the estimates of TVI-ESPRIT and ECC-TVI-ESPRIT algorithms are consistent with the target directions, while other algorithms can only efficiently estimate the signal directions at $60^{\circ }$ and $-75^{\circ }$, which reflects the advantage of TVI-ESPRIT and ECC-TVI-ESPRIT algorithms in the omnidirectional DOA estimation. On the other hand, the estimates from the Toeplitz-ESPRIT algorithm show significant deviation from the target directions, especially at $-75^{\circ }$, while other velocity-independent algorithms accurately estimate the directions, which demonstrates that the cross-array have the advantage of acoustic velocity-independence.

### 3.2. Performance Comparison Under Different SNR

In this set of experiments, the performance of the proposed ECC-TVI-ESPRIT algorithm is compared with other algorithms at different SNR. The root mean square error (RMSE) is used as a measure of accuracy. For each DOA estimation algorithm, *W* independent Monte Carlo simulations are conducted. The angle estimate of the *k*th signal in the *w*th (w=1,…,W) simulation is denoted as ${\hat{\theta }}_{k,w}$. The RMSE for the algorithm is defined as:

(28) $$\mathrm{RMSE}=\sqrt{\frac{1}{WK}\sum_{k=1}^{K}\sum_{w=1}^{W}{\left({\hat{\theta }}_{k,w}-\theta_{k}\right)}^{2}}$$

**Figure 3:**
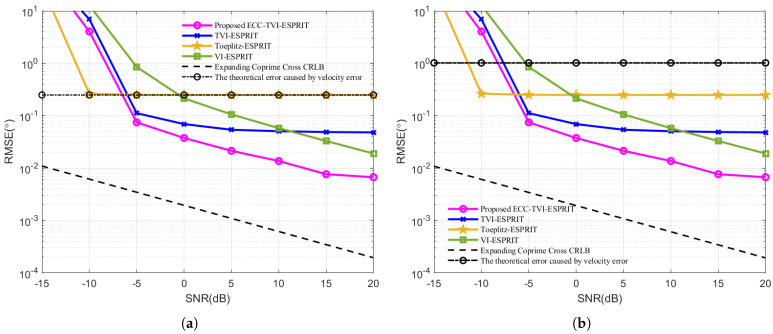
RMSE under different SNR: (**a**) Δc=5 m/s. (**b**) Δc=20 m/s.

By comparing the two simulation results shown in Figure 3, it is evident that the RMSE curves of the VI-ESPRIT, TVI-ESPRIT, and ECC-TVI-ESPRIT algorithms show little variation, while the RMSE curve of the Toeplitz-ESPRIT algorithm experiences a large shift, and it almost coincides with the theoretical error curve caused by the acoustic velocity error when SNR is large. This indicates that the acoustic velocity errors significantly affect the estimation accuracy of the Toeplitz-ESPRIT algorithm, whereas the VI-ESPRIT, TVI-ESPRIT, and ECC-TVI-ESPRIT algorithms are less impacted. The ECC-TVI-ESPRIT algorithm outperforms the other three algorithms in environments with inaccurate acoustic velocity, which proves the effectiveness of the proposed algorithm in improving the performance. Meanwhile, the estimation accuracy of the ECC-TVI-ESPRIT algorithm is higher than the TVI-ESPRIT algorithm, which proves the effectiveness of the low-rank matrix filling technique in interpolating the missing values in the Toeplitz matrix. In addition, it can be observed that at low SNR levels, the RMSE curve is not parallel to CRB, indicating the presence of bias in the estimation.

### 3.3. Performance Comparison Under Different Number of Snapshots

This experiment presents the relationship between the RMSE and the number of snapshots for the Toeplitz-ESPRIT, VI-ESPRIT, TVI-ESPRIT, and ECC-TVI-ESPRIT algorithms.

**Figure 4:**
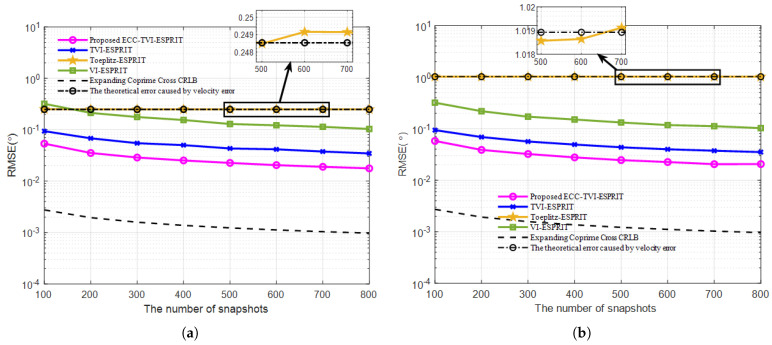
RMSE under different number of snapshots: (**a**) Δc=5 m/s. (**b**) Δc=20 m/s.

Comparing Figure 4a,b, the RMSE curves of the Toeplitz-ESPRIT algorithm are greatly shifted, while the curves of the other three velocity-independent algorithms do not change significantly. This demonstrates that the ECC-TVI-ESPRIT algorithm has the advantage of acoustic velocity-independence. Moreover, as the number of snapshots increases, the estimation accuracy of the Toeplitz-ESPRIT algorithm does not improve and almost coincides with the theoretical error curve, which indicates that the impact of acoustic velocity error is the main source of error in this algorithm. The estimation accuracy of the ECC-TVI-ESPRIT algorithm is better than the TVI-ESPRIT algorithm regardless of the snapshot conditions and the presence of the acoustic velocity error. This further confirms the high-precision advantage of the ECC-TVI-ESPRIT algorithm and the effectiveness of the “low-rank matrix filling” method in filling the “signal holes” of the differential array.

### 3.4. Performance Comparison Under Different Acoustic Velocity Errors

In this experiment, the RMSE versus sound speed error relationship is plotted for the four algorithms. The range of sound speed error is set from −20 m/s to 20 m/s, with an interval of 5 m/s.

**Figure 5:**
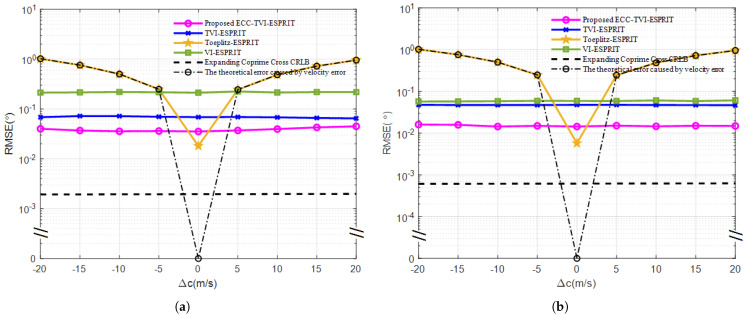
RMSE under different acoustic velocity errors: (**a**) SNR=0 dB. (**b**) SNR=10 dB.

The experimental results in Figure 5 show that the RMSE curves of the VI-ESPRIT, TVI-ESPRIT, and ECC-TVI-ESPRIT algorithms are close to straight lines, which indicates that their performance is almost unaffected by the acoustic velocity error. However, the RMSE curve of the Toeplitz-ESPRIT algorithm increases as the magnitude of acoustic velocity error |Δc| grows, which indicates that the Toeplitz-ESPRIT algorithm based on the linear array is greatly affected by the accuracy of the acoustic velocity. Finally, the estimation accuracy of the ECC-TVI-ESPRIT algorithm is further improved compared with the TVI-ESPRIT algorithm, which shows that the ECC-TVI-ESPRIT algorithm is effective in filling the vacant terms of the Toeplitz matrix and contributes to the improvement in estimation accuracy.

## 4. Conclusions

In this paper, an underwater velocity-independent DOA estimation algorithm based on improved Toeplitz matrix reconstruction is proposed. The algorithm uses two cross-expanded coprime arrays to eliminate the acoustic velocity factor. On the one hand, it possesses the advantages of acoustic velocity-independent and omnidirectional estimation compared to other ESPRIT algorithms (e.g., Toeplitz-ESPRIT). On the other hand, compared with other velocity-independent algorithms (e.g., VI-ESPRIT and TVI-ESPRIT), the proposed algorithm uses the low-rank matrix filling technique to generate data for the “signal holes”, which provides more degrees of freedom and further improves the accuracy of the DOA estimation. Finally, the simulation results verify the effectiveness of the proposed algorithm and its superiority over other algorithms. This study does not account for mutual coupling effects between sensors. Although mutual coupling may introduce perturbations in practical implementations, previous research [19] has developed effective compensation algorithms. Integrating mutual coupling mitigation into our framework could be an interesting direction for future work.

## Data Availability

Data will be made available upon request.
